# Exciton dynamics of C_60_-based single-photon emitters explored by Hanbury Brown–Twiss scanning tunnelling microscopy

**DOI:** 10.1038/ncomms9461

**Published:** 2015-09-29

**Authors:** P. Merino, C. Große, A. Rosławska, K. Kuhnke, K. Kern

**Affiliations:** 1 Max-Planck-Institut für Festkörperforschung, Heisenbergstraße 1, Stuttgart 70569, Germany; 2 Ecole Polytechnique Fédérale de Lausanne, Lausanne 1015, Switzerland

## Abstract

Exciton creation and annihilation by charges are crucial processes for technologies relying on charge-exciton-photon conversion. Improvement of organic light sources or dye-sensitized solar cells requires methods to address exciton dynamics at the molecular scale. Near-field techniques have been instrumental for this purpose; however, characterizing exciton recombination with molecular resolution remained a challenge. Here, we study exciton dynamics by using scanning tunnelling microscopy to inject current with sub-molecular precision and Hanbury Brown–Twiss interferometry to measure photon correlations in the far-field electroluminescence. Controlled injection allows us to generate excitons in solid C_60_ and let them interact with charges during their lifetime. We demonstrate electrically driven single-photon emission from localized structural defects and determine exciton lifetimes in the picosecond range. Monitoring lifetime shortening and luminescence saturation for increasing carrier injection rates provides access to charge-exciton annihilation dynamics. Our approach introduces a unique way to study single quasi-particle dynamics on the ultimate molecular scale.

## Abstract (web summary)

Electrons and holes trapped in a molecular crystal couple to form excitons. Here, the authors use scanning tunnelling microscopy to inject current with submolecular precision into structural defects in solid C_60_ and demonstrate single photon emission from the excitons trapped there.

## Graphical abstract

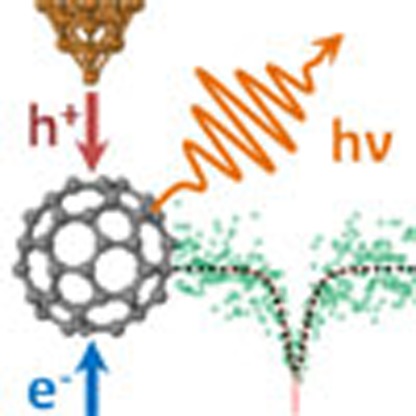

Exciton spectroscopy plays a crucial role in studying the lowest electronic excitations in semiconductor nanostructures near their band edge and allows addressing excited state lifetimes and decay channels^1^,^2^,^3^. For applications in photonics^4^,^5^, the exciton dynamics in quantum-confined systems is important, as it allows to manipulate the train of emitted photons. Measurements of photon–photon time correlations on such emitters can be employed to prove their sub-Poissonian photon statistics for which short delays between successive emission events are less likely to occur than longer ones (photon ‘antibunching'). This property of individual quantum systems, such as molecules^6^,^7^, quantum dots^8^,^9^ or crystal defects^10^,^11^, is of importance for possible applications as single-photon (SP) emitters, which are key elements for cryptographic code transmission^12^ and form the basis for a further development of photon-on-demand light sources^13^. The key point is the use of individual emitters as non-classical photon statistics becomes obscured in ensemble measurements. Many sub-Poissonian light emitters have been demonstrated to date in which diluted dye molecules are driven by optical (laser) fields^14^. In recent years, elaborate current-driven emitters have also emerged^7^,^15^. In these cases, the suppression of photons from adjacent emitters requires spatially or energetically well-separated emission centres (ECs) and low background fluorescence. By contrast, it is possible to address individual emitters even at high densities if a selective excitation by charge injection is employed^16^,^17^. This has the potential to access classes of SP sources, which could not be investigated otherwise. Addressing recombination with sub-wavelength resolution has been established by using scanning optical near-field probes^14^,^18^,^19^,^20^, stimulated emission depletion microscopy^21^ and, prominently, by electroluminescence from organic molecules in scanning tunnelling microscopy (STM)^22^,^23^. However, the ultimate goal of accessing exciton dynamics in sub-Poissonian emitters with sub-molecular resolution remains a challenge.

In this article, we demonstrate a solution to this problem by using STM to inject current with sub-molecular precision and a Hanbury Brown–Twiss (HBT) interferometer to confirm SP emission in electroluminescence. We profit from the efficient (tip-enhanced) coupling of the local electromagnetic emission in the tunnel junction to detect the light in the far field^24^. Controlled injection allows us to generate a bound state of an electron and a hole (an exciton) and let them interact with an additional charge during their radiative decay. The interaction between excitons and electrons can result in exciton annihilation, a process with fundamental and technological relevance in photon-exciton-energy conversion^25^,^26^.

## Results

### Electroluminescence of C_60_ ECs

**Figure 1:**
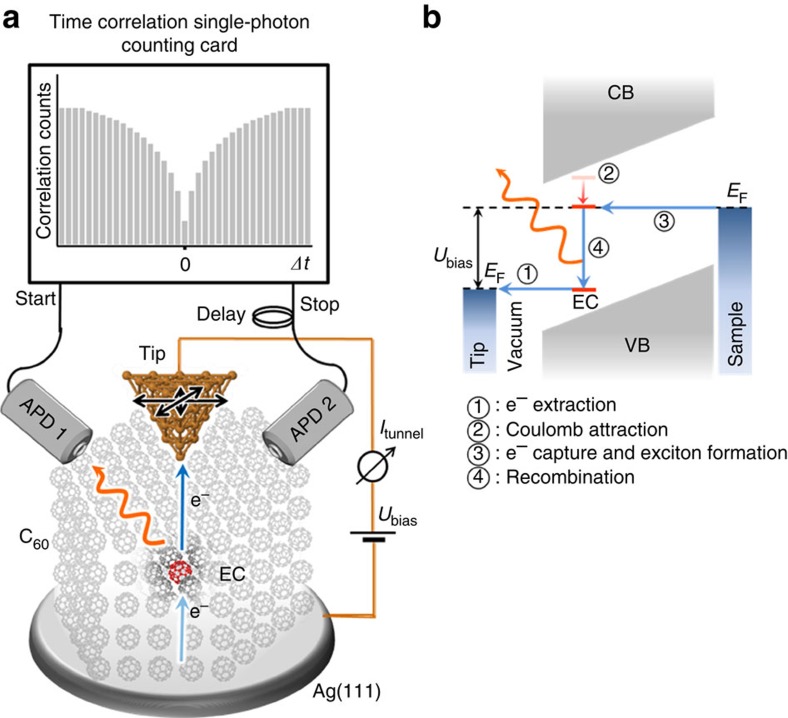
Scheme of the experimental setup of a scanning tunnelling microscope (STM) extended by a Hanbury Brown–Twiss (HBT) interferometer and energy diagram of the luminescence process. (**a**) The STM tip extracts electrons from a C_60_ molecule at the surface of the film. Extraction conditions are controlled by the tip position (*x*,*y*) as well as the applied voltage *U*_bias_ and the extracted tunnel current *I*_tunnel_. As the hole is trapped at the emission centre (EC), it can capture an electron from the substrate and form an exciton. The recombination event can be detected by one of the two time-resolving photon counters (APD: avalanche photo diode). One APD operates as a timer-start and the other one as a timer-stop for a time-correlated single-photon counting card. The distribution of measured start-stop times *Δt* provides the second-order photon correlation function g^(2)^(*Δt*). For sub-Poissonian light sources, for example, a single-photon emitter, events at smaller |*Δt*| values occur less often than events at larger |*Δt*| ; *g*^(2)^(*Δt*) shows a minimum at *Δt*=0. (**b**) Energy level scheme and electron flow within the C_60_ multilayer containing an EC. The detailed mechanism is described in the text. CB, conduction band; VB, valence band.

Figure 1a shows the integration of the HBT interferometer scheme into our low temperature STM design. We couple two time-resolved SP counters each to one optical path leading independent of the others to the STM junction situated in ultra-high vacuum (UHV)^27^. A third optical path (not shown) is coupled to a spectrograph. All optical detectors operate under ambient conditions. For further details of the experimental setup see ref. 27. We study the electroluminescence from individual ECs on epitaxially grown crystalline C_60_ films (6–10 monolayers (ML)) on Ag(111). In contrast to excitations of single-molecules, the electron–hole pairs in organic crystals are typically described as excitons^1^. The thickness of the film is an important parameter as lower layers decouple the higher layers and avoid quenching of excitonic emission by the metal substrate^28^,^29^,^30^; we observe luminescence from ECs for films thicker than ∼5 ML.

**Figure 2:**
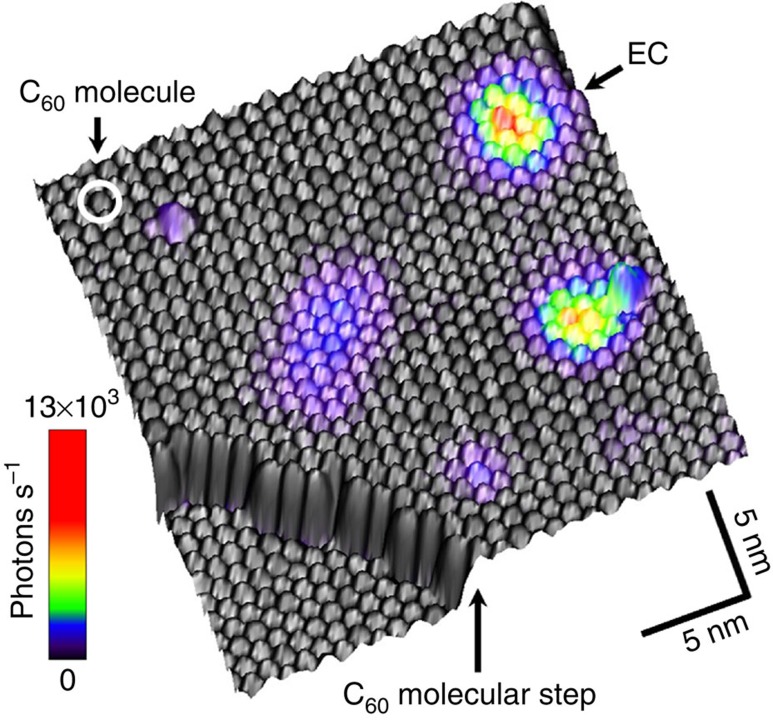
Three-dimensional topographic STM image of the C_60_ surface overlaid with the simultaneously obtained electroluminescence photon map. The colour represents the detected light intensity. Several emission centres (ECs) of different size and intensity can be identified. Arrows indicate the positions of an EC, a C_60_ molecule within the surface layer, and a 0.8nm high C_60_ crystal step (image size: 25 × 25 nm^2^, *U*_bias_=−3.0 V, *I*_tunnel_=30 pA).

In Fig. 2, we present several ECs in a STM topograph overlaid with the simultaneously acquired luminescence intensity (colour scale; see Supplementary Fig. 1 for details). The topograph is recorded by scanning the tip at constant current of 30 pA over the surface while recording simultaneously the height and light intensity at every point. The light intensity has been converted to a false colour scale so that colour spots indicate the ECs positions where enhanced light emission occurs. This map can be interpreted analogously to a photoluminescence map exchanging the excitation with a laser with charge injection from a sharp tip. ECs exhibit strong STM-induced luminescence and appear isolated from each other. The size and luminescence intensity vary from centre to centre with yields up to 10^−4^ detected photons per electron (we estimate 15% instrumental detection efficiency). The background emission from the C_60_ multilayer is at least two orders of magnitude lower. For typical experimental conditions (*U*_bias_=−3.2 V, *I*_tunnel_≤1,000 pA), the centres do not bleach and remain stable over arbitrarily long time (>10 h).

**Figure 3:**
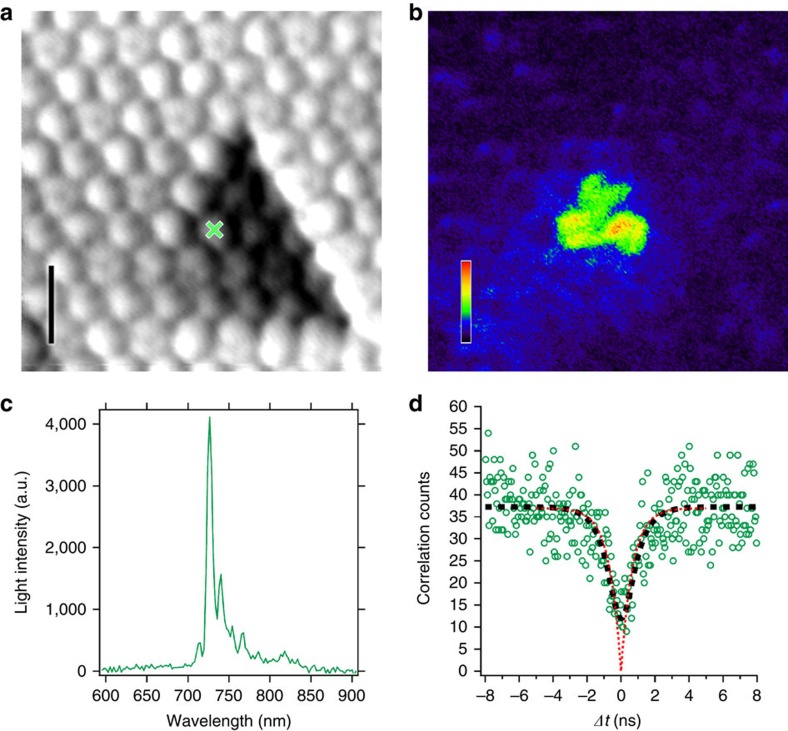
Nanoscale characterization of a single-photon emission centre. (**a**) Topographic STM image, *U*_bias_=−3.2 V, *I*_tunnel_=51 pA; scale bar, 2 nm. (**b**) Simultaneously recorded electroluminescence photon map, the false colour scale bar represents the light intensity 0–67 kcounts s^−1^. (**c**) Electroluminescence optical spectrum recorded on the molecule marked by the green cross in **a** (120 s, *U*_bias_=−3.2 V, *I*_tunnel_=500 pA). (**d**) Photon correlation (green open circles) measured with the HBT interferometer during electron extraction on the molecule marked by the green cross in **a**; integration time 3 h, time correlated single-photon detection channel width 50 ps, *U*_bias_=−3.2 V, *I*_tunnel_=51 pA. The dashed black line is a best fit based on the characteristics of a single-photon source convoluted with the detector time resolution. The dotted red line represents the same fit after removing the detector broadening.

In Fig. 3 we fully characterize one EC appearing near a dislocation. Figure 3a shows the submolecularly resolved topographic STM image. The simultaneously obtained photon map (Fig. 3b) reveals that emission can be excited locally with high yield only on three molecules. The luminescence spectrum (Fig. 3c) exhibits an intense peak around 730 nm accompanied by a weaker line on the blue and a series of lines on the red side attributed to a vibrational progression^23^. A recent theoretical publication identified the small satellite peak on the blue side as a fingerprint of singlet exciton fluorescence from C_60_ (ref. 31). The observed peak wavelength and line structure resemble STM-induced luminescence spectra attributed to C_60_ exciton recombination, which have been reported for thin (2-4 layers) C_60_ nanocrystals decoupled from the substrate by a thin NaCl layer^29^. We find by tunnelling spectroscopy (see Supplementary Fig. 2 and Supplementary Note 1 for details) that the strong localized luminescence on our much thicker films originates from shallow charge traps at or close to the surface. Disorder in molecular crystals can induce shallow trap states inside the bandgap^32^ pinning the position of exciton formation and decay. This may relate to the so-called X-traps reported in photoluminescence studies of C_60_ single crystals^33^,^34^. In our experiments, structural and orientational disorder in the molecular films naturally form the shallow traps after room temperature sample growth and rapid cool-down inside the 4K microscope. ECs due to disorder may exist in the surface layer or deeper within the C_60_ film, which rationalizes the different emission morphologies and intensities.

We explain the emission from ECs by means of the energy level diagram in Fig. 1b. ECs consist of an electron and a hole trap. Below a certain bias voltage threshold, an electron can tunnel from the occupied state of the defect to the tip (process ①). This charges the hole trap positively and lowers the upper electron trap state below the Fermi energy of the substrate (process ②). Once an electron tunnels into that upper electron trap state while the lower state is still unoccupied (process ③), an exciton is created. At last, exciton recombination (process ④) results in photon emission. This luminescence mechanism leads to sub-Poissonian emission statistics measurable with a HBT interferometer. The obtained photon–photon correlation histograms (Fig.1a) are proportional to the second-order correlation function g ^(2)^(*Δt*) in the low count rate limit. For a perfect SP emitter, no coincidences occur at zero time delay: two photons are never emitted simultaneously^8^.

### C_60_-based SP emission

Figure 3d shows the photon correlation measurement of the EC as raw data in units of correlation events per 50 ps time bin (3 h integration). At delay time zero, it exhibits a reduction of 67% of the uncorrelated events. Values above 50% exclude two or more photon emitters and prove the SP nature of the light source. Similar photon antibunching curves were obtained on all the ECs investigated. Electrically driven photon correlation studies in STM have been published earlier but only positive correlations (photon bunching) have been reported^35^. We thus demonstrate that SP emission can be realized in a STM-induced luminescence setup. The correlation minimum does not reach zero because of the limited detector time resolution (full-width at half-maximum=1.2 ns, see Supplementary Fig. 3 and Supplementary Note 2 for details). Correcting for this instrumental broadening, we obtain (red dotted curve in Fig. 3d) an exponential rise from time zero with a characteristic time constant of 0.69 ns.

## Discussion

**Figure 4:**
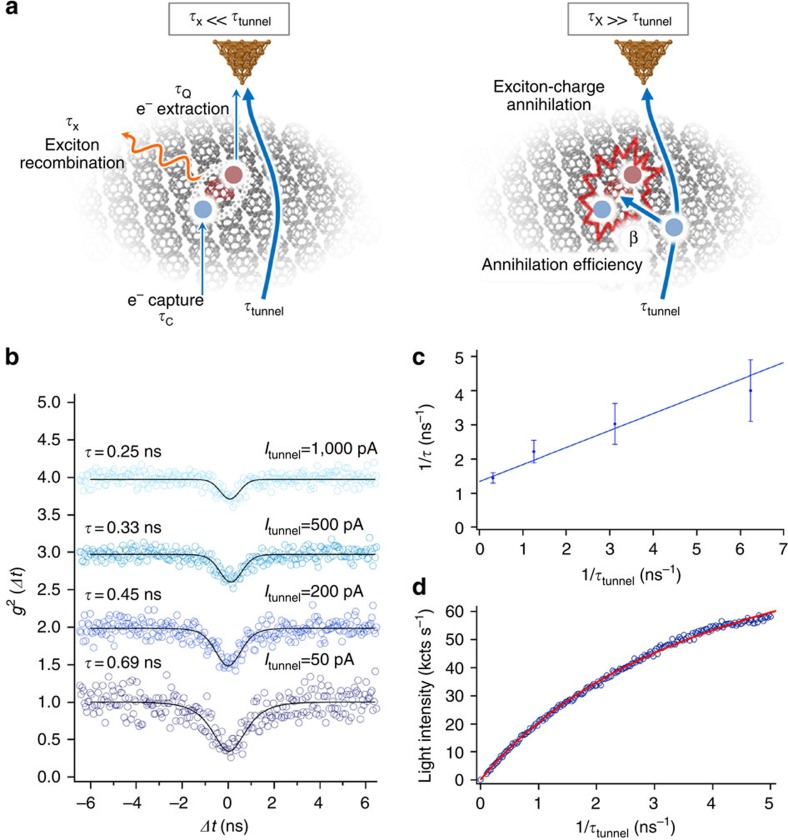
Exciton-charge annihilation and three-state model. (**a**) Graphical representation of the three-states discussed in detail in the text. (**b**) Blue circles: second-order correlation function *g*^2^ (*Δt*) measured for the currents indicated on the right-hand side (*U*_bias_=−3.2 V). The three upper curves have been upshifted for clarity by multiples of one. The black lines are best fits for a single-photon emission convoluted by the detector time resolution function (full-width at half-maximum=1.2 ns). The values on the left-hand side are the time constants from the corresponding fits. (**c**) Inverse measured time constants plotted as a function of current (in units of elementary charge per nanosecond). The interception with the vertical axis yields the true exciton lifetime without charge–exciton interaction. The positive slope quantifies the efficiency of charge exciton annihilation, see equation (1). The error bars represent the precision (systematic error) of the lifetime after correction for the measured detector resolution. (**d**) Blue circles: Photon intensity in one avalanche photo detector versus tunnel current (*U*_bias_=−3.0 V). Red line: best fit to the three-state model including exciton charge annihilation according to equation (2).

We describe the luminescence dynamics within a three-state kinetic model based on the processes depicted in Fig. 1b and schematically presented in Supplementary Fig. 4. When an electron is extracted from the highest filled band of the molecular film by the STM tip, it will almost instantaneously be replaced by an electron from the substrate. There is, however, a probability *α*<<1 to create an exciton (Fig. 4a). This requires that the created hole will reside in the charge trap of the EC. There, the hole may be refilled in a slower process (detrapping) or may capture an electron from the substrate, thus, create an exciton at the EC site. Our observation of the highly localized exciton emission proves the existence of exciton formation via electron capture on the EC position. The electron capture time *τ*_C_ is on a picosecond scale because of the low tunnel barrier of the film and the strong electrostatic attraction of the trapped hole. Once formed, the exciton can decay to the ground state with its proper decay constant *τ*_X_ and may emit a photon which triggers a pulse in one of the detectors. The third relevant time constant, the time for successful hole trapping *τ*_Q_, which discounts the events where detrapping occurs, is substantially longer.

When the current is increased (Fig. 4b), the minimum of the second-order correlation function g^(2)^(*Δt*) becomes narrower and less deep. Accounting again for the detector broadening, we find a continuous reduction of the observed lifetime (*τ*) for increasing current (values listed in Fig. 4b). To describe this behaviour, we include the exciton charge annihilation process as a non-radiative decay channel in the three state model (Fig. 4a). Then, the measured lifetime (*τ*) is given by:


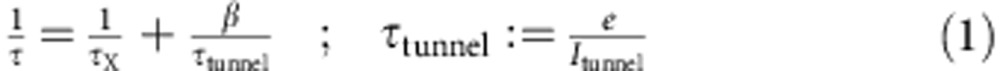


with *e* being the elementary charge and 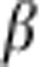 the exciton charge annihilation efficiency. Figure 4c displays a linear fit of the inverse *τ* as a function of the inverse *τ*_tunnel_ yielding an unperturbed exciton lifetime of *τ*_X_=0.75 ns from the interception with the vertical axis. The positive slope of the curve reflects the annihilation efficiency and from the fit of the data series in Fig. 4b we obtain *β*=0.50±0.12. This means that an exciton is quenched with 50% probability by the next injected charge. Whether the charge carrier transfers energy and dissociates the exciton or its charge induces non-radiative quenching cannot be concluded from our data.

The model is quantitatively supported by the observed emission saturation as a function of current. The measurement in Fig. 4d provides independent evidence for the high charge-exciton annihilation probability at the ECs. Assuming *τ*_C_<<*τ*_x_, *τ*_Q_ and a small exciton creation efficiency (*α*), we can approximate the photon emission *E* as a function of current:


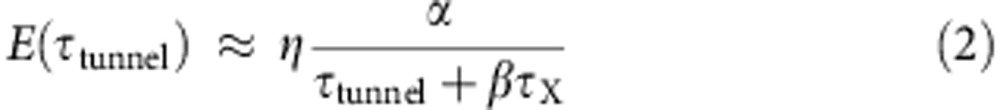


with *η*<<1 expressing the transfer probability from exciton decay to photon detection (see Supplementary Note 3 for details). We obtain *β*=0.3±0.1 from the fit of the data shown in Fig. 4d (see Supplementary Fig. 5 for details). The minimalistic three-state model including exciton-charge annihilation thus describes our observations completely. The two methods employed to determine *β* both yield quenching efficiencies in the order of unity and suggest that exciton charge annihilation is the dominant decay channel in the regime *τ*_tunnel_<<*τ*_x_. The deviation in *β* may be reduced by experiments (for example, pump-probe pulse experiments) providing a precise measurement of the electron capture time (*τ*_C_), which is presently below our time resolution. In addition, *ab-initio* calculations^36^ addressing exciton–charge interactions could also be of interest for a deeper understanding of the quenching mechanism.

In conclusion, we introduce a local probe technique to study the dynamics of the formation and annihilation of electron–hole pairs at the molecular scale. Structural defects in C_60_ films form individual electrically addressable SP emitters. At higher currents, the charge-exciton annihilation cuts in on the observed lifetime. We may speculate that both, antibunching and annihilation, are in fact related: The efficient quenching of an exciton by the next charge will prohibit any biexciton formation and will ensure SP emission. This could be exploited as an alternative way to create SP emitters with tunable decay time based on a different mechanism than the conventional two-level systems. Moreover, by STM manipulation, it might be possible to create shallow traps deliberately. This would enable designing nanometre-precise SP emitter structures, which could be used to study quantum interference phenomena or even entanglement. In general, the HBT-STM combination applied in this letter opens new pathways in exciton engineering and nanophotonics and may be used to study charged and neutral particles and their interaction in other organic semiconductors and other materials (for example, transition metal dichalcogenides), as well as more exotic quasiparticles like Rydberg excitons^2^, trions^37^ or biexcitons.

## Methods

### Sample fabrication

The samples were prepared under UHV conditions and introduced *in situ* into the STM. The Ag(111) single-crystal substrate was cleaned by repeated cycles of Ar ion sputtering and annealing to 800 K. The 6–10 monolayer C_60_ (purity 99.9%) thin film was subsequently evaporated from a Knudsen source while keeping the substrate at room temperature.

### Scanning tunnelling microscope

The experiments are performed with an in-house built, low-temperature (4.2 K), UHV (<10^–11^ mbar) STM. Light originating from the tunnel junction is collected by *in situ* optics cooled to the same temperature as the whole STM assembly. The light is guided to a photon counter or spectrometer outside UHV without rise of the sample temperature inside a liquid helium cryostat. The setup allows operating the HBT setup and the spectrometer simultaneously. The spectrometer is an Acton SP 300i with a 150 lines per mm blazed (500 nm) grating coupled to a Peltier-cooled intensified CCD camera. For further information about the numeric aperture of the lenses and the geometry of the optical setup, see ref. 27.

### HBT interferometer

Photon–photon time correlation measurements were carried out using a gold tip on individual ECs. As their emission spectrum is dominated by one line, no spectral discrimination of the light in front of the photon counting detectors has been used. The experimental setup is sketched in Fig. 1a. The light emission from the tunnel junction is monitored from two orthogonal directions. The photons are detected by two SP counting avalanche photo diodes (Single-photon counting module SPCM-AQRH-14, supplier: Perkin-Elmer).The start–stop intervals between detected photons are recorded using a time-correlation SP counting PC card (Time-correlated single-photon counting PC card SPC-130, supplier: Becker&Hickl). The correlation data were measured with STM current-feedback on. Individual measurements were integrated over minutes and summed up to yield integration times of hours. The dark count rate of the detectors is 70 counts s^−1^, which leads to a negligible contribution in the correlation data.

## Additional information

**How to cite this article:** Merino, P. *et al*. Exciton dynamics of C_60_-based single-photon emitters explored by Hanbury Brown–Twiss scanning tunnelling microscopy. *Nat. Commun.* 6:8461 doi: 10.1038/ncomms9461 (2015).

## Supplementary Material

Supplementary Information Supplementary Figures 1-5, Supplementary Notes 1-3 and Supplementary References
