# Supplementary material for: Exciton dynamics of C60-based single-photon emitters explored by Hanbury Brown–Twiss scanning tunnelling microscopy
Source: Nat Commun. 2015 Sep 29;6:8461. doi: 10.1038/ncomms9461 (PMC4598842; doi:10.1038/ncomms9461)
Supplement: Supplementary Information — Supplementary Figures 1-5, Supplementary Notes 1-3 and Supplementary References [file ncomms9461-s1.pdf]

## Supplementary Figures

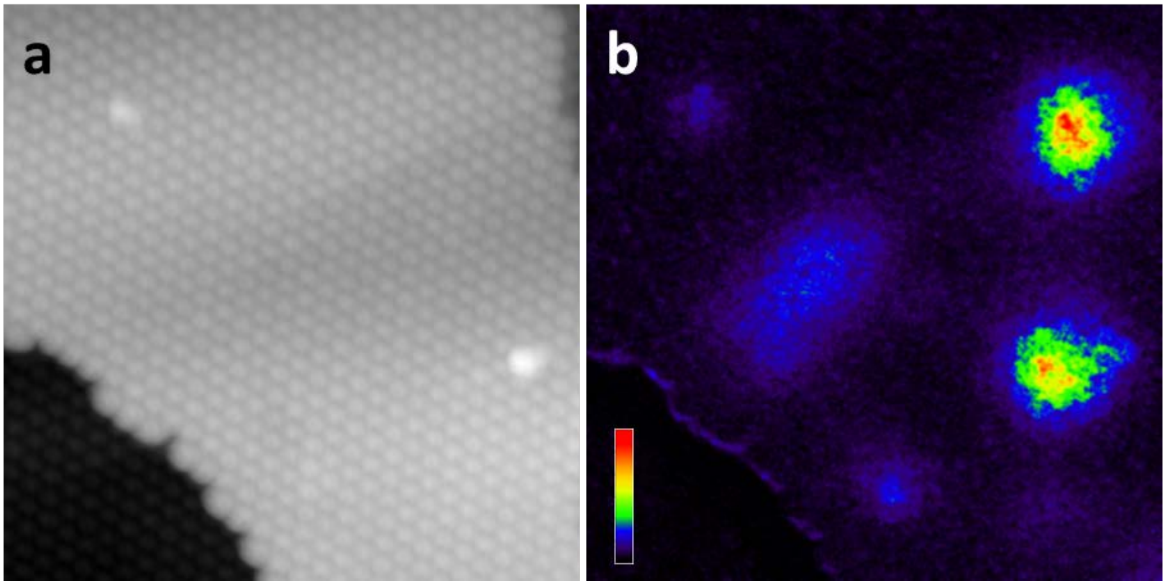


***Supplementary Figure 1**: **Independent tunneling and photon channels of Figure 2. a)*** *STM topograph of $C_{60}$ a multilayer. In Fig. 2 we overlaid the photon map on the 3D representation of the scanning tunneling microscopy (STM) topographic micrograph. Here we present the independent maps. The emission centers (ECs) appearing on this region are related to defects different from the one presented in Fig. 3. While the latter is associated to a screw dislocation, the ones presented here are rather not visible in the topography map or appear as rotational disorder within the layers. A single molecular step appears running in the lower-left part of the image.* ***b)*** *Electroluminescence photon map obtained simultaneously with the topography map introduced in a); scale bar represents photon intensity 0-13 kcts $s^{-1}$. Image size: 25x25 $nm^2$ $U_{bias}$ =-3.0 V, $I_{tunnel}$ =30 pA.*

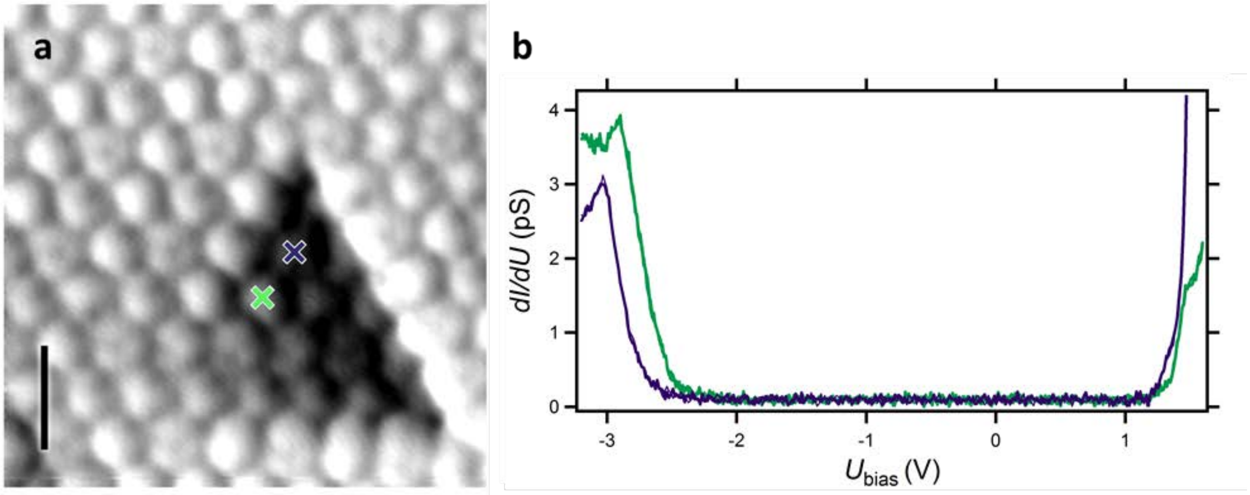


***Supplementary Figure 2**: **Scanning tunneling spectroscopy of an emission center. a)*** *STM topograph of the single photon emission center discussed in the Fig.3 of the main text. The green and blue crosses mark the position where the dI/dU curves were obtained.* $U_{bias}$ *=-3.2 V,* $I_{tunnel}$ *=51 pA, scale bar 2 nm.* ***b)*** *dI/dU tunneling spectroscopic spectra obtained in the marked positions.*

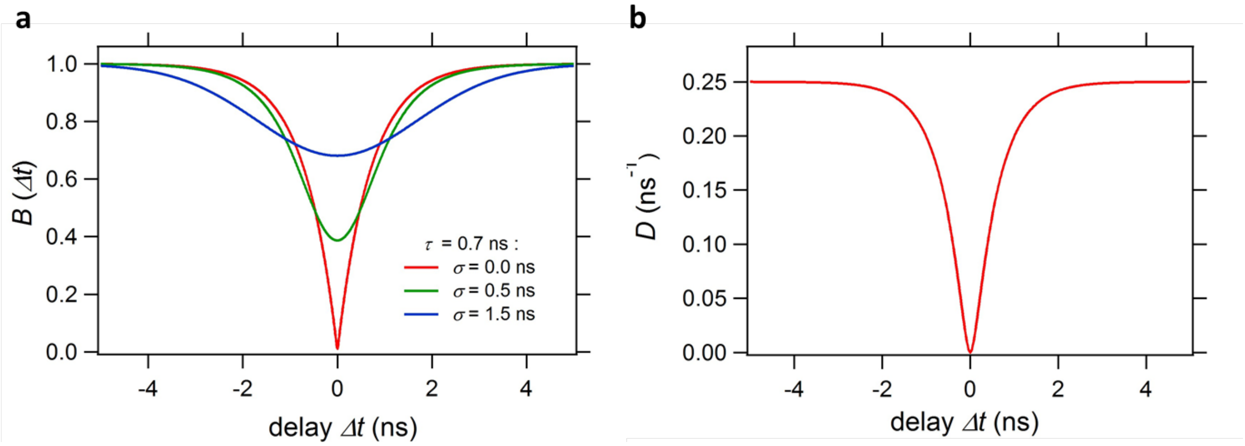


***Supplementary Figure 3**: **Plot of the function B and D for the indicated parameters. a**) The FWHM of the Gaussian is given by $\sqrt{8\,ln(2)}\,\sigma = 2.36\,\sigma$. The green and the blue curves thus correspond to a detector FWHM of 1.2 ns and 3.5 ns, respectively. The green curve is close to the experimental condition. **b)** Function D from eq. (6) for the parameter set: $k_1$= (3.2ns)$^{-1}$, $k_2$= 10 ns$^{-1}$, $k_3$= (0.7ns)$^{-1}$.*

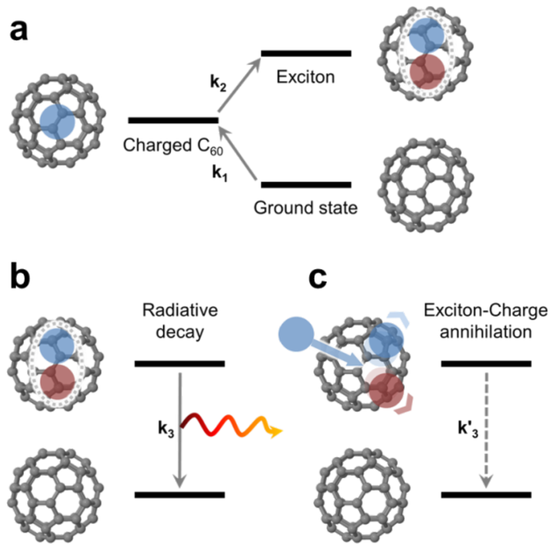


***Supplementary Figure 4**: **Scheme of the 3 state model used to interpret our experimental results. a)*** *The three states (ground state, charged $C_{60}$ and exciton) are introduced and the rate constants leading to exciton formation ($k_1$ and $k_2$) are introduced .* ***b)*** *Radiative decay from the excited state to the ground state has a rate fonstant of $k_3$.* ***c)*** *Exciton-charge annihilation decay from the excited state to the ground state is introduced in the model though the rate constant $k'_3$.*

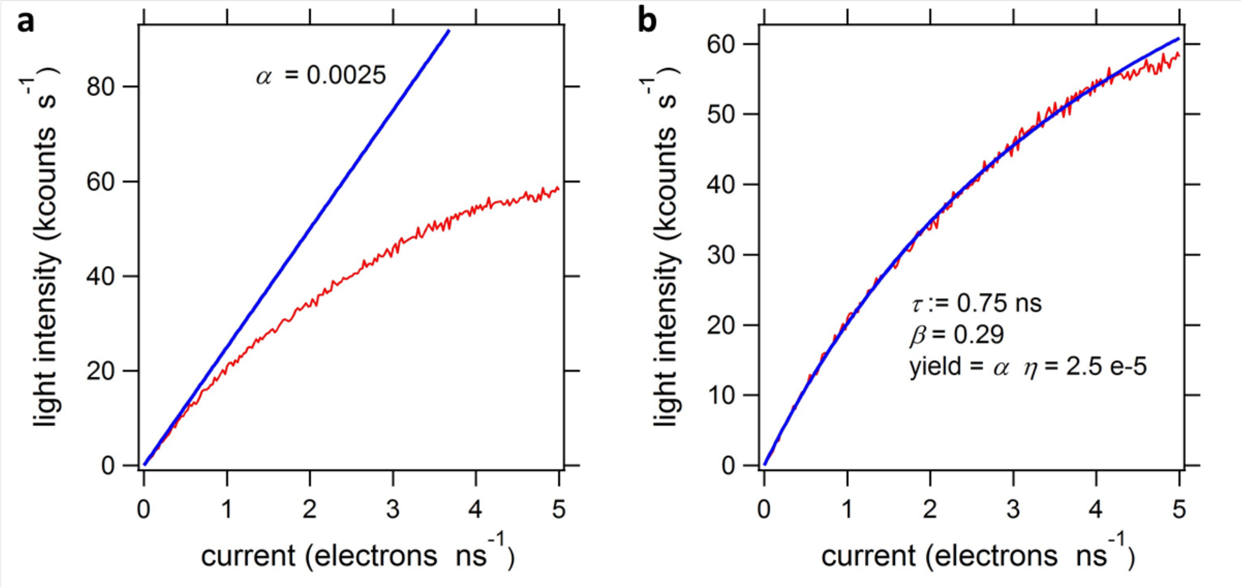


***Supplementary Figure 5**: **Fitting of the luminescence saturation without and with exciton charge annihilation. a)*** *Fit without exciton charge annihilation. Function $E(I_{\text{tunnel}})$ from eq. (8) plotted for the parameters $k_1 = \alpha I$, $k_2 = 10\ ns^{-1}$, $k_3 = (0.75ns)^{-1}$, $\eta = 10^{-2}$.* ***b)*** *Fit of the luminescence vs. current with exciton charge annihilation. The experimental data from Fig. 4e of the main text can be fitted very well already in the approximation (12) for $\alpha \cdot \eta = 2.5 \cdot 10^{-5}$ and $\beta = 0.29$ .*

## Supplementary Note 1: Scanning tunneling spectroscopy

In Supplementary Figure 2 we introduce two scanning tunneling spectra (dI/dU) obtained on the single photon emission center characterized in Fig. 3 of the main text. The spectra presented in Supplementary Figure 2 b were obtained on top of the positions marked with crosses of their respective colors in Supplementary Figure 2 a. The green spectrum was obtained on one of the three molecules that excite with high yield photon emission in the dislocation. The blue was obtained on a non-emitting molecule.

The green spectrum presents a filled electronic state shifted into the bandgap by 0.2 eV with respect to the blue spectrum. This state inside the bandgap is the cause for the strong emission obtained when electrons are extracted from that molecule and responsible for the single photon emission. The splitting off of the hole trap is typically much more pronounced than the split off of the electron trap. The spectra qualitatively corroborate the energy level diagram presented in the main text. Spatial mapping of the defective charge trap states in ECs will be published elsewhere.

We would like to comment on the difference between the band gap observed in Supplementary Figure 2 b and the luminescence photon energy of 1.7eV. Two corrections have to be applied. The thick dielectric $C_{60}$ layer penetrated by the strong electric field in the STM leads to a significant shift of observed electronic states with respect to their true (field free) energies. We found experimentally that under the given tunneling parameters the apparent widening of the band gap with respect to the true gap amounts to an increase of roughly 8% for each additional $C_{60}$ layer. Comparison of the value in Supplementary Figure 2 b with our data obtained for a double $C_{60}$ layer on Ag(111) (apparent gap 2.8eV) is thus in good agreement with an assumed thickness of 8 $C_{60}$ layers in our study. The remaining difference between 2.8eV and the classical literature value of 2.3eV [1] results from the remaining field shift within the last two layers. The second correction comes from the known exciton binding energy (electron-hole attraction) of 0.46eV.[2] Finally, a rather small residual contribution of 0.2eV-0.3eV may be attributed to the exciton trapping in the ECs.

## Supplementary Note 2: Obtaining the life time from the correlation measurements:

The antibunching of sub-Poissonian emitters is described by a model containing only ground and excited state by the simple exponential recovery with the recovery time $\tau$ [3]:

$$A\,(\Delta t) \;=\; 1 - \,e^{-\frac{|\Delta t|}{\tau}} \tag{1}$$

Models containing additional intermediate states may provide deviations from this simple shape. The broadening of function A in (1) by a normalized Gaussian detection function can be calculated analytically:

$$B(\Delta t) \;=\; \frac{1}{\sqrt{2\pi\sigma^2}} \int_{-\infty}^{\infty} e^{-\frac{(\Delta t-\zeta)^2}{2\sigma^2}} \left(1 - \,e^{-\frac{\zeta}{|\tau|}}\right) d\zeta \;=$$

$$= 1 - \frac{1}{2}\, e^{\frac{\sigma^2}{2\tau^2}} \cdot \left( e^{-\frac{\Delta t}{\tau}} \cdot \left(1 - erf\left\{\frac{1}{\sqrt{2}}\left(\frac{\sigma}{\tau} - \frac{\Delta t}{\sigma}\right)\right\}\right) + e^{\frac{\Delta t}{\tau}} \cdot \left(1 - erf\left\{\frac{1}{\sqrt{2}}\left(\frac{\sigma}{\tau} + \frac{\Delta t}{\sigma}\right)\right\}\right)\right) \qquad (2)$$

where *erf* (x) is the error function. Function B with the known Gaussian width of 1.2ns FWHM ($\sigma$ = 0.5 ns) could be used to fit the experimental $g^{(2)}(\Delta t)$ curves (Fig.3d) in the main text and to obtain from these fits the recovery times $\tau$. See Supplementary Figure 3a for examples with various $\sigma$.

The data in the paper is evaluated and fitted by going even one step further. We employed the photon correlation of the two detectors in response to ps light pulses from a spectrally filtered (690nm) supercontinuum light source. The measured correlation function which is due to the detector characteristics could be described very well by the sum of two Gaussians with different heights and widths. With this approximation it is again possible to obtain the analytical convolution of (1) because the convolution of a sum of two functions is simply the sum of the two convoluted functions. The numerical fit of the analytical function to the measured data is straight forward and directly yields the recovery time $\tau$ in the experiment which are the ones we present in the paper.

## Supplementary Note 3: Emission intensities and photon correlations in the three-state Model:

Correlation experiments in photo-excitation require a minimum of two states to account for the behavior: ground state and excited (singlet exciton) state. Including an additional long-lived excited state (e.g. triplet exciton) [4] leads to a three-state model (see Supplementary Figure 4).

The electrical excitation requires a minimum of 3 states because the generation of an exciton cannot be achieved in 1 step as in optical excitation but requires the successive creation of a hole and then the capturing of an electron at the monitored site. In the discussed experiments the electron extraction by the tip always precedes the electron capture so that a fourth (trapped electron) state will not be considered. The population of the trap by a hole is assumed to be linear in the tunnel current $I_{\text{tunnel}}$ so that the first rate constant (the inverse of the time constant) of the model is given by:

$$k_1 = \alpha^{-1} \cdot \frac{I_{\text{tunnel}}}{e} \qquad (3)$$

In the model we regard, however, only those trapped holes, which are converted into an exciton because a separate detrapping process is not included. $\alpha$ is thus the exciton creation probability for each charge injected by the STM tip.

The capture of an electron requires the pre-existence of a trapped hole since due to the energetic position of electronic states a negatively charged trap lies too high in energy. The existence of a negatively charged trap under typical experimental conditions can be excluded since this electron could tunnel to the STM tip and emit a photon in an inelastic tunneling process thus emitting a broad plasmonic light spectrum, which is, in fact, not observed.

The electron capture by the trapped hole is fast (<< 1ns) as discussed in the main text. Its rate $k_2$ is assumed to be constant. If a weak dependence on the current exists, we would expect that a higher current would slightly increase the electron capture due to the increased driving force of the electric fields. The experimental observation is, however, opposite why we neglect the dependence of $k_2$ on current.

$$k_2 = const \gg \frac{1}{1\,ns} \tag{4}$$

The decay of the exciton occurs by its proper time constant $\tau_X$

$$k_3 = \frac{1}{\tau_X} \tag{5}$$

As shown in an earlier publication, this rate constant may contain already non-radiative quenching e.g. by the nearby metal electrodes. [5] This and another publication [6] suggest a life time of the lowest singlet exciton of the order of 1 ns.

The rate equation model developed up to here can be solved analytically for the time-dependent occupation numbers of the three states: $n_g(\Delta t)$, $n_c(\Delta t)$, $n_X(\Delta t)$. When a recombination has taken place at time zero (initial condition of the solution), the probability for another exciton recombination at time delay $\Delta t$ is obtained as:

$$D\,(\Delta t) = k_3\; n_X(\Delta t) \;= \frac{P}{R}\,\left(1 + \frac{S-Q}{2\,Q}\cdot e^{-\frac{S+Q}{2}t} - \frac{S+Q}{2\,Q}\cdot e^{-\frac{S-Q}{2}t}\right) \tag{6}$$

with the substitutions

$$P = k_1 \cdot k_2 \cdot k_3 \;\; ; \;\; S = k_1 + k_2 + k_3 \;\; ; \;\; R = k_1\,k_2 + k_2\,k_3 + k_3\,k_1 \;\; ; \;\; Q = \sqrt{S^2 - 4\,R} \;\; . \qquad (6')$$

The curve in Supplementary Figure 3b exhibits the characteristic recovery time of anti-bunching as 0.62 ns which is close although not identical to the inverse rate constant $k_3$. The smallest of the rate constants, $k_1$ , enters into the intensity but does not appear as a time constant. Rate constant $k_2$ appears through a small parabolic section at the minimum at time-zero (compare to minimum of the red curve in Supplementary Figure 3a). This parabolic section is absent in two-state models and is expected to become measurable only for high enough detector time resolution and excellent correlation statistics.

The detected correlation rate is much lower than obtained by *D* due to various losses. These comprise a constant fraction of non-radiative recombination, finite coupling to plasmonic light emission and finally the optical transmission and detection efficiencies. We summarize the transmission remaining after all these losses in the constant $\eta$ so that the experimental correlation rate can be described by:

$$F\,(\Delta t) = \eta\;k_3\;n_X(\Delta t) \;= \eta\,\frac{P}{R}\,\left(1 + \frac{S-Q}{2\,Q}\cdot e^{-\frac{S+Q}{2}t} - \frac{S+Q}{2\,Q}\cdot e^{-\frac{S-Q}{2}t}\right) \tag{7}$$

To account for the measured quantum yield of $2.5 * 10^{-5}$ photons/electron we assume an exciton creation efficiency of $\alpha = 2.5*10^{-3}$ excitons/charge and a detection loss factor $\eta = 10^{-2}$. This value of $10^{-2}$ is based on the estimated optical transmission of the optical line (ca 15% for optical transmission and detection [7]) and the roughly 5% efficiency of plasmonic free space emission. [8] A more precise separation of the measured quantum yield into the two factors cannot be provided here as the two factors cannot be independently determined in the experiment.

From (7) we obtain the time-averaged photon intensity E in one detector as a function of tunnel current:

$$E\,(I_{\mathrm{tunnel}}) = \lim_{\Delta t\to\infty} F\,(\Delta t) = \eta\,\frac{P}{R} = \eta\,\frac{k_1 \cdot k_2 \cdot k_3}{k_1\,k_2 + k_2\,k_3 + k_3\,k_1} \tag{8}$$

which we plot in Supplementary Figure 5a for similar parameters as used in Supplementary Figure 3b. We find that the function is dominated by a linear dependence on the current and that deviations from linearity would occur only if $k_1$ becomes comparable to $k_3$ and $k_2$.

Eqns. (7) and (8) do not yet include the current-dependent exciton life time reduction. We introduce parallel to process $k_3$ the non-radiative charge-induced exciton quenching of the exciton by $k_3{}'$. This process is linear in the current with a charge exciton annihilation efficiency $\beta$:

$$k_3{}' = \beta^{-1} \cdot \frac{I_{\mathrm{tunnel}}}{e} \tag{9}$$

This modification turns the list of substitution parameters (6’) into

$$P = k_1 \cdot k_2 \cdot k_3 \;; \qquad S = k_1 + k_2 + k_3 + k_3{}' \;;$$

$$R = k_1\,k_2 + (k_2 + k_1)\,(k_3 + k_3{}') \;;\; Q = \sqrt{S^2 - 4\,R} \tag{10}$$

and the current dependent light emission (8) into:

$$E\,(I_{\mathrm{tunnel}}) = \eta\,\frac{k_1 \cdot k_2 \cdot k_3}{k_1\,k_2 + (k_2 + k_1)\,(k_3 + k_3{}')} \tag{11}$$

We conclude with a simplified result by introducing two approximations. We assume (*) that the electron capture process $k_2$ is by far the fastest of the three processes and that (**) the exciton creation efficiency $\alpha$ is much smaller than 1. Then we obtain

$$E\,(I_{\mathrm{tunnel}}) \approx^{(*)} \eta\,\frac{k_1 \cdot k_3}{k_1 + k_3 + k_3{}'} \approx^{(**)} \frac{\eta\,\alpha}{\tau_{\mathrm{tunnel}} + \beta\,\tau_X} \tag{12}$$

wherein we substituted for simplicity $\tau_{\mathrm{tunnel}} = \frac{e}{I_{\mathrm{tunnel}}}$ , the average time between two tunneling charges. As $\tau_{\mathrm{tunnel}}$ is given by the tunnel current and $\tau_X$ is known from the time resolved correlation data, this formula has only two free parameters: The electroluminescence quantum yield $\alpha * \eta$ which is the slope near zero current and the annihilation quantum efficiency $\beta$ which accounts for the deviation from linearity. In Supplementary Figure 5b we plot the best fit to the experimental data using eq. (12).

We shall comment that for larger injection rates (i.e. tunneling currents) the photon count rate theoretically completely levels off and even can decline. However, reaching these experimental conditions is a difficult task since high tunneling currents result in more unstable tips, and eventually in crashes destroying the ECs. However exploring these high current ranges can be of interest in future investigations.

## Supplementary References:


1 Lof, R. W., van Veenendaal, M. A., Koopmans, B., Jonkman, H. T. & Sawatzky, G. A. Band gap, excitons, and Coulomb interaction in solid $C_{60}$. *Phys. Rev. Lett*. **68**, 3924-3927 (1992).

2 Dresselhaus, M. S., Dresselhaus, G. & Eklund, P. C. Science of fullerenes and carbon nanotubes: their properties and applications. *Academic press,* page 691 (1996).

3 Michler, P. et al. Quantum correlation among photons from a single quantum dot at room temperature. *Nature* **406**, 968-970 (2000).

4 Basché, T., Moerner, W. E., Orrit, M. & Talon, H. Photon antibunching in the fluorescence of a single dye molecule trapped in a solid. *Phys. Rev. Lett.* **69**, 1516-1519 (1992).

5 Kuhnke, K., Becker, R., Epple, M. & Kern, K. $C_{60}$ Exciton Quenching near Metal Surfaces. *Phys. Rev. Lett.* **79**, 3246-3249 (1997).

6 Long, J. P., Chase, S. J. & Kabler, M. N. Photoelectron spectroscopy and dynamics of excitons in $C_{60}$ and photopolymerized $C_{60}$ films. *Chem. Phys. Lett*. **347**, *29-35*, (2001).

7 Kuhnke, K. et al. Versatile optical access to the tunnel gap in a low-temperature scanning tunneling microscope. *Rev. Sci. Instrum.* **81**, (2010).

8 Wang, T., Boer-Duchemin, E., Zhang, Y., Comtet, G. & Dujardin, G. Excitation of propagating surface plasmons with a scanning tunnelling microscope. *Nanotechnology* **22**, 175201 (2011).
